# Long-term outcomes of a pediatric HIV treatment program in Maputo, Mozambique: a cohort study

**DOI:** 10.3402/gha.v8.26652

**Published:** 2015-08-17

**Authors:** Jan Walter, Lucas Molfino, Verena Moreno, Celeste G. Edwards, Mafalda Chissano, Angels Prieto, Tatiana Bocharnikova, Annick Antierens, Johnny Lujan

**Affiliations:** 1 Médecins Sans Frontières, Maputo, Mozambique; 2 Ministry of Health, Maputo, Mozambique; 3 Médecins Sans Frontières, Geneva, Switzerland

**Keywords:** HIV, pediatric HIV care, task shifting, Mozambique, anthropometric scores, CD4 cell count

## Abstract

**Objective:**

To describe long-term treatment outcomes of a pediatric HIV cohort in Mozambique.

**Design:**

Retrospective analysis of routine monitoring data.

**Setting:**

Secondary health care facilities in the Chamanculo Health District of Maputo.

**Subjects:**

A total of 1,335 antiretroviral treatment (ART) naïve children <15 years of age enrolled in HIV care between 2002 and 2010.

**Intervention:**

HIV care, ART (since 2003), task shifting to lower cadre nurses, counseling by lay counselors, active patient tracing, nutritional support, support by a psychologist, targeted viral load testing, and switch to second-line treatment.

**Main outcome measures:**

Kaplan–Meier estimates for retention in care (RIC), CD4 cell percentage, body mass index for age z-score, and adjusted incidence rate ratios for attrition (death or loss to follow-up) as calculated by Poisson regression.

**Results:**

The RIC at 6 years in the pre-ART cohort was 44% (95% confidence interval: 38–49), and the one at 8 years in the ART cohort was 70% (64–75). Risk factors for attrition included young age, low CD4 percentage, underweight, active tuberculosis, and enrollment/treatment initiation after 2006. The mean CD4 percentage increased strongly at 1 year on treatment and remained high thereafter. The body mass index for age z-score sharply increased at 1 year after treatment initiation before stabilizing at pre-ART levels thereafter.

**Conclusions:**

Good clinical and immunological treatment outcomes up to 8 years of follow-up on ART can be achieved in a context of shortage of health workers and a high level of task-shifting approach.

HIV treatment of children can be more complicated than that of adults, since HIV infection in children presents more aggressively; treatment requires continuous adaptation of drug doses to weight; clinicians need to be trained in pediatrics; and treatment delivery may depend on caregivers who are sick with AIDS themselves. In addition, diagnostics among infants is complicated due to the necessity of viral RNA or DNA detection, which is much less available than serological diagnosis. Given these potential difficulties in pediatric HIV care, it is necessary to demonstrate that successful treatment of HIV-infected children in resource-limited settings can be achieved (1).

Mozambique has one of the highest HIV prevalences in sub-Saharan Africa, which is estimated to be 11.5%, 15–49 years of age. Among adolescents (12–14 years), the prevalence is estimated to be 1.8%, in children (0–11 years) 1.4%, and in infants (under 1 year) 2.3% (2). In 2012, there were an estimated 1.6 million persons living with HIV in Mozambique, including 180,000 children. An estimated 77,000 persons died from AIDS in 2012 alone (www.unaids.org). In addition to the HIV epidemic, Mozambique is also one of the least developed countries in the world according to the human development index (www.undp.org), and it is facing a severe human resource crisis in the health care sector (3).

There are only few reports on treatment outcomes of pediatric HIV cohorts from Mozambique (4–6), and – to our knowledge – none with long-term follow-up. We, therefore, conducted a retrospective analysis of routine monitoring data collected from a cohort of HIV-infected children treated in Maputo. The main aim of this study was to describe long-term outcomes and to identify risk factors associated with loss to follow-up and mortality that would allow implementing future improvement of pediatric HIV care in Maputo and in similar settings.

## Methods

### Study design

We conducted a retrospective analysis of routine monitoring data collected from a cohort of HIV-infected children enrolled in the HIV treatment program in the Chamanculo Health District between the initiation of the data collection in 2002 and the start of the decentralization process of pediatric care to primary health care centers in the district in 2010. All ART naïve HIV-infected children below 15 years of age at enrollment were included in the analysis. The children were followed until the end of 2012, when the cohort has been decentralized to primary health care centers.

### Setting

Since 2001, Médecins Sans Frontières (MSF) has been supporting the Mozambican Ministry of Health in the care and treatment of HIV-infected patients in the Chamanculo Health District, in Maputo, which serves an estimated target population of 329,872 inhabitants. As secondary health care facilities, this area included, until 2009, two day hospitals (Alto-Maé and Chamanculo, the latter being a pediatric clinic), which were subsequently replaced by the Chamanculo Hospital. In October 2009, following the closure of the day hospitals, a Reference Center in Alto-Maé was created in order to ensure access to specialized care for HIV-infected patients with complications (including those with low CD4 counts, advanced clinical stage, Kaposi Sarcoma, and those requiring second- and third-line ART).

Between 2001 and 2007, HIV-infected children were enrolled in HIV care together with their mothers (The Prevention of Mother-to-Child Transmission (PMTCT) program has been in place since 2002) at the Chamanculo and the Alto-Maé day hospitals. Systematic data collection was started in 2002. ART became available in 2003. By the end of June 2007, all children from Alto Maé day hospital had been transferred to the pediatric HIV clinic at the Chamanculo Hospital, where they were followed until further decentralization to primary health care centers in 2012.

### Study population

Among 18,497 entries of HIV-infected patients in the project databases, 29 (0%) entries were excluded due to missing or implausible dates of birth or dates of the first visit, 16,753 (91%) entries since they belonged to patients 15 years or older at the day of enrollment, 68 (0%) entries since they were from patients enrolled after 2010, and 236 (1%) entries since they were from patients transferred within the program and could be merged to already existing entries. Among the resulting 1,411 entries, 76 (5%) patients were excluded since they were already on ART at enrollment in the program, resulting in a final study cohort of 1,335 HIV-infected, ART naive children below 15 years of age.

### Patient management

Patients were managed in accordance with Mozambican and World Health Organization (WHO) guidelines. They were diagnosed with HIV using virological or serological methods depending on their age, received treatment of opportunistic infections, cotrimoxazole prophylaxis, were monitored by laboratory investigations, and received ART when clinically or immunological indicated. The standard first-line ART regimen was stavudine or zidovudine plus lamivudine plus nevirapine or efavirenz. A pediatric fixed-drugs combination was introduced in 2008. The suspicions of treatment failure were based on clinical, immunological, and virological criteria. Second-line ART regimens included a boosted protease inhibitor plus two new nucleotide reverse transcriptase inhibitors that were adjusted according to the children's age and the availability of pediatrics formulations. Patients in need of second-line ART were transferred out to receive this at a separated (also MSF-supported) reference site.

As in many other resource-constrained countries, the provision of care in the program was mainly ensured by non-physician clinicians (nurses and clinical officers), who were especially trained in pediatric clinical care. Most clinical tasks of follow-up were conducted by lower cadre nurses. Close program supervision was conducted by a referent physician who was also in charge of ART initiation and diagnosis, and the treatment of complicated cases.

Follow-up visits were scheduled monthly for patients receiving ART and for those less than 1 year of age, otherwise these visits were bimonthly. At each visit, clinical data were documented by clinicians on standardized forms. CD4 cell count and/or percentage were monitored every 6 months. Viral load measurements, even though not routinely performed, were used to confirm suspicion of treatment failure.

The program had a strong psychosocial component that included educational/counseling sessions on health and hygiene (including on nutrition, breastfeeding, treatment adherence, side effects, and toxicities), educational games and stories for children, a trained lay counselor, who provided pre- and post-HIV testing and counseling, pre-ART counseling, adherence counseling, and pediatric HIV disclosure sessions. Psychological support was provided by a referent psychologist when needed. Referral for social support to respective programs and organization of support groups for mothers and caretakers as well as for disclosed adolescents was part of the program. An active tracing system for patients who failed to attend clinic appointments (‘defaulter tracing’) was also set up. This included telephone reminders and home visits if not responsive to those calls; adherence follow-up by home visits and psychological supports at home were also carried out.

Care before 2007 involved a nutritional program, which provided milk formula and accessories for safe formula preparation to children above 6 months of age. Additional nutritional support included the provision of ready to use therapeutic food and formula to children in need after 2007. A buffer stock for antiretrovirals (ARVs) and drugs for opportunistic infections was also introduced.

Tuberculosis (TB) screening was done by standard questionnaire using the Crofton score which was adjusted over the years. If TB was suspected, microscopic analysis was conducted on sputum samples. Gastric lavage and X-ray were performed as needed. Patients needing special care or hospitalization were transferred to a tertiary hospital in Maputo.

### Data collection

The national paper-based system was used to collect data related to HIV activities in the program. Data were subsequently entered in the electronic database for follow-up and care for HIV and AIDS (FUCHIA, Epicentre, Paris). An Access database was used to collect data about defaulter tracing. Project documentation was reviewed, and key staff interviewed to reconstruct the history of the program.

Data were exported from the databases in November 2013.

### Ethical consideration and reporting

The study followed the rules for a waiver for review by the MSF international ethics review board and patient consent, due to the use of routine data for retrospective data analysis.

The STROBE guidelines for reporting of observational studies were followed (7).

### Statistical analysis

Body mass index for age z-score (BAZ) was calculated using STATA macros downloaded from the WHO (www.who.int). They were calculated separately for children <5 and ≥5 years of ages and subsequently combined into one variable. Biologically implausible values for z-scores, as flagged by the macros, were excluded from the analysis. Among the available anthropometric z-scores, we chose to include BAZ in the analysis, since it could be calculated for all ages and is most closely related to underweight, which previously was identified as a risk factor for mortality among children (8). Age was categorized in standard age groups of below 18 months, 18–59 months, and above 59 months. Year of enrollment/treatment initiation was grouped into three categories with similar numbers of patients.

Baseline CD4 cell counts and percentages were included in the analysis, if they were conducted within 90 days of the first visit or treatment initiation. For the follow-up, CD4 cell counts/percentages as well as height and weight were included if they were collected within 90 days of annual follow-up time point.

To analyze the retention in care (RIC), we used Kaplan–Meier methods. The time until death in days was calculated by subtracting the date of death from the date of enrollment or the date of treatment initiation as appropriate. The time until loss to follow-up was calculated by subtracting the data of the last visit from the date of enrollment or date of treatment initiation. In concordance with national guidelines, patients were defined as loss to follow-up if their last scheduled appointment had been missed for more than 60 days. All the remaining observations were censored at the transfer-out date or on 31th of December 2012 for three children without a transfer-out date in the database. Observations in the pre-ART cohort were censored at the date of ART initiation. Kaplan–Meier plots for the pre-ART cohort were truncated at 6 years and for the ART cohort at 8 years, due to small numbers thereafter.

Since the proportional hazard assumption in Cox-regression models was violated, we chose Poisson regression for the analysis of factors associated with the incidence rate of mortality or loss to follow-up. We included CD4 percentages in this analysis, as this was more closely related to the outcomes than CD4 cell count. Missing CD4 cell percentages, WHO stages, and BAZ were imputed with the cohort means (CD4 cell count) or zeros for categorical variables and controlled for by adding dummy variables specifying patients with missing data. Covariates were retained in adjusted analysis, if they were significantly associated with the outcome.

All analysis was conducted using STATA/SE 9.0 for Windows (College Station, TX, USA).

## Results

### Baseline characteristics

The 1,335 children included in the analysis had a median age at enrollment of 3.7 years. Fifty-seven percent of them were in WHO stage III or IV and nearly a quarter with CD4 percentage <10%. Five percent of the children had active TB, and 15% were underweight as indicated by a BAZ <−2. As expected, at treatment initiation children were slightly older and a higher proportion of them was in advanced WHO stage or had a CD4 cell percentage below 10% (Table 1).

**Table 1:** Characteristics of HIV-infected children at program enrollment and at ART initiation, Chamanculo District, Maputo, Mozambique

	At enrollment	At treatment initiation
*N*	1,335	833
*n* (%) live outside Maputo Cidade	394 (31)	242 (30)
*n* (%) female	669 (50)	400 (48)
Age [median years (IQR)]	3.7 (1.7, 6.7)	4.4 (2.1, 7.8)
<18 months [*n* (%)]	290 (22)	142 (17)
18–59 months [*n* (%)]	537 (40)	310 (37)
59+ months [*n* (%)]	508 (38)	381 (46)
WHO stage III or IV [*n* (%)]	645 (57)	591 (74)
CD4 cell count [median (IQR)]^a^	668 (342, 1072)	514 (262, 968)
CD4 percentage [median (IQR)]^a^	16 (10, 22)	13 (9, 18)
<10% [*n* (%)]	223 (24)	208 (29)
10–20% [*n* (%)]	415 (44)	370 (52)
≥20% [*n* (%)]	307 (32)	135 (19)
BMI for age z-score [median (IQR)]^b^	−0.4 (−1.5, 0.4)	−0.3 (−1.3, 0.5)
>−2 [*n* (%)]	650 (83)	522 (86)
−2 to −3 [*n* (%)]	71 (9)	48 (8)
<−3 [*n* (%)]	66 (8)	39 (6)
*n* (%) active TB at enrollment/treatment initiation	85 (6)	67 (8)
Year of enrollment/ treatment initiation [*n* (%)]		
2002–2006	538 (40)	206 (25)
2007–2008	399 (30)	271 (33)
2009–2012	398 (30)	356 (43)
Median (IQR) years of follow-up	0.2 (0.1, 0.8)	3.4 (2.1, 4.2)

IQR=interquartile range; due to missing data the numbers shown may not tally to the total; TB=tuberculosis.

a CD4 counts were available for 945 (71%) patients at enrollment and 713 (86%) at treatment initiation.

b BMI for age z-scores were available for 787 (59%) patients at enrollment and 609 (76%) at treatment initiation.

### Treatment outcomes

Of the 1,335 children included in the analysis, 46 (3%) died and 304 (23%) were lost to follow-up before they initiated ART; 833 (62%) children initiated ART. The remaining 152 (11%) children were either transferred out (96 [7%]) to other districts or to hospitals of higher complexity or were decentralized within the district (56 [4%]) before ART initiation. Of the children, who initiated ART, 49 (6%) died during the follow-up, 122 (15%) were lost to follow-up, and 233 (28%) were transferred out. The transferred group included 32 (4%) children who were receiving second-line treatment at the referral center in the district at the end of 2012. The remaining 429 (52%) children were decentralized to the primary health care centers from the health area, until the end of 2012.

**Fig. 1:**
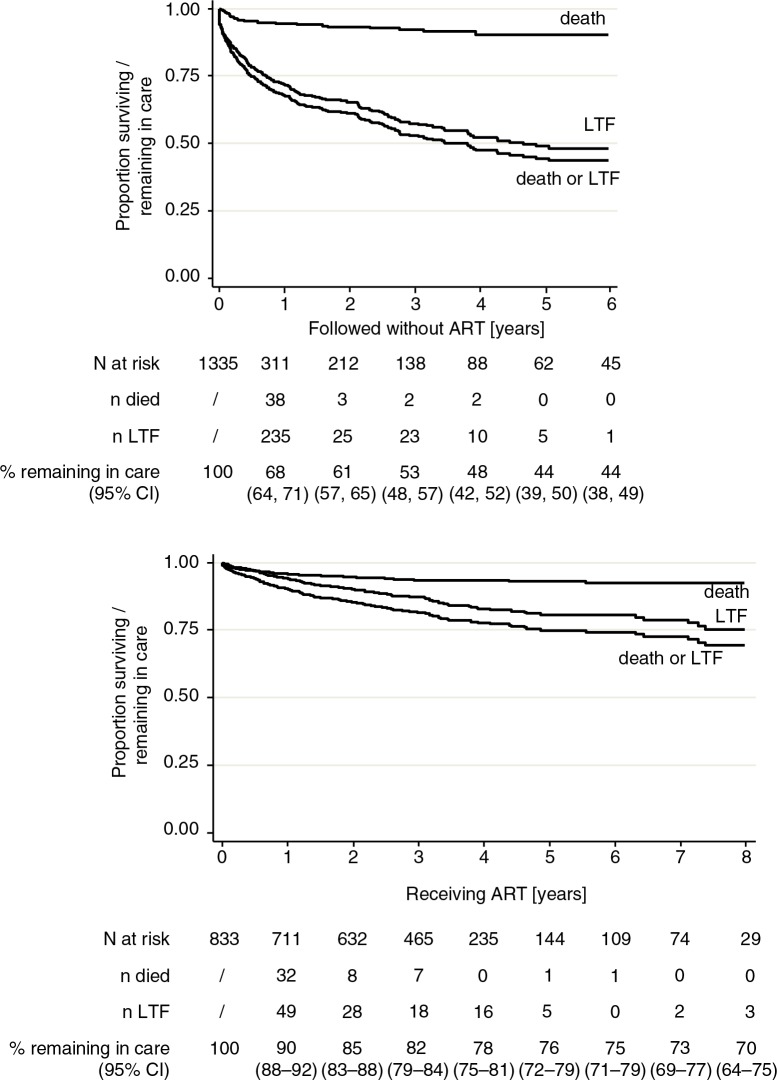
Retention in care of HIV-infected children in the Chamanculo District of Maputo, Mozambique. Shown are Kaplan–Meier plots for the time until death or loss to follow-up (LTFU) before (upper panel) and after (lower panel) ART initiation. The table below the graphs shows the numbers at risk, the number of persons died, the number of persons lost to follow-up, and the Kaplan–Meier estimates for the RIC. The LTFU and deaths do not tally to the total due to transfer out of some patients.

Using Kaplan–Meier methods, the RIC after 1 year in the pre-ART cohort was 68% (95% confidence interval [CI]: 64–71). It then declined more or less steadily to 44% (95% CI: 38–49) at 6 years. Among patients on ART, it was 90% (95% CI: 88–92) after 1 year, after which it declined to 70% (95% CI: 64–75) at 8 years (Fig. 1).

The total follow-up time was 1,235 person years (py) before ART initiation and 2,791 py thereafter. The attrition rate (death or loss to follow-up) was 28.3 (95% CI: 25.5–31.5) per 100 py in the pre-ART cohort and 6.1 (95% CI: 5.3–7.1) per 100 py in the ART cohort (Table 2). The attrition rate was highest in the first 3 months of follow-up in both, the pre-ART as well as the ART cohort (Fig. 1).

**Table 2:** Rates of outcomes before and after ART initiation, Chamanculo District, Maputo, Mozambique

	Before ART initiation	After ART initiation
Person years (py) of follow-up	1,235	2,791
*n* deaths	46	49
*n* LTFU	304	122
Mortality [events/100 py (95% CI)]	3.7 (2.8, 5.0)	1.8 (1.2, 2.3)
LTFU [events/100 py (95% CI)]	24.6 (22.9, 27.5)	4.4 (3.7, 5.2)
LTFU or mortality [events/ 100 py (95% CI)]	28.3 (25.5, 31.5)	6.1 (5.3, 7.1)

ART=antiretroviral treatment; CI=confidence interval; LTFU=loss to follow-up.

### Factors associated with mortality

The main biomedical risk factors for death in the pre-ART and ART cohort were young age (<1.5 years) and underweight as indicated by a BAZ <−2 (Table 3). Some of these associations were remarkably strong. For example in the pre-ART cohort, the adjusted incidence rate ratio [IRR] for below 1.5 years of ages as compared to ≥5 years was 6.9 (95% CI: 2.8–17.2), the one for BAZ <−2 versus BAZ ≥2 even 9.3 (4.0–22.0). In the ART cohort, the adjusted IRR for young age was 2.8 (1.3–5.8) and for BAZ <−2: 4.7 (2.4–9.1). Additional strong biomedical risk factors in adjusted analysis were active TB at ARV treatment initiation (IRR: 3.4, 95% CI: 1.7–7.1) and low CD4 percentage at enrollment (IRR per 10% increase: 0.5, 95% CI: 0.3–0.8). In both cohorts, enrollment or treatment initiation before 2006 was associated with significantly better outcomes.

**Table 3:** Poisson regression modeling factors associated with death or loss to follow-up for pediatric pre-ART and ART cohorts in the Chamanculo Health District, Maputo, Mozambique, 2002–2012

	Before ART initiation				After ART initiation			
	
	Mortality		Loss to follow-up		Mortality		Loss to follow-up	
			
	Unadjusted IRR (95% CI)	Adjusted IRR (95% CI)	Unadjusted IRR (95% CI)	Adjusted IRR (95% CI)	Unadjusted IRR (95% CI)	Adjusted IRR (95% CI)	Unadjusted IRR (95% CI)	Adjusted IRR (95% CI)
Female			0.8 (0.7, 1.0)					
Age [years]								
<1.5	**7.1 (3.2, 15.8)**	**6.9 (2.8, 17.2)**	1.2 (0.9, 1.6)		**3.6 (1.9, 7.1)**	**2.8 (1.3, 5.8)**	**2.1 (1.3, 3.5)**	**1.8 (1.1, 2.9)**
1.5 to 5	1.4 (0.6, 3.3)	2.1 (0.8, 5.4)	0.9 (0.7, 1,1)		1.0 (0.5, 2.0)	1.5 (0.7, 3.2)	1.4 (0.9, 2.0)	1.3 (0.9, 2.0)
≥5	Reference	Reference	Reference		Reference	Reference	Reference	Reference
WHO stage III/IV	**2.7 (1.4, 5.2)**		1.0 (0.8, 1.3)		1.4 (0.7, 2.8)		0.9 (0.6, 1.3)	
CD4 percentage per 10% increase	**0.4 (0.2, 0.6)**	**0.5 (0.3, 0.8)**	**0.8 (0.6, 1.0)**		1.0 (0.7, 1.4)		1.0 (0.8, 1.3)	
BMI for age z-score <−2	**18.8 (8.5, 41.5)**	**9.3 (4.0, 22.0)**	**3.3 (2.0, 5.4)**	**3.6 (2.2, 5.9)**	**5.8 (3.1, 10.7)**	**4.7 (2.4, 9.1)**	1.5 (0.9, 2.7)	
Active TB	1.5 (0.6, 3.8)		0.7 (0.5, 1.2)		**3.6 (1.8, 7.0)**	**3.4 (1.7, 7.1)**	**1.7 (1.0, 3.0)**	**2.1 (1.2, 3.7)**
Year of enrollment or treatment initiation								
2002–2006	**0.3 (0.1, 0.6)**	**0.1 (0.0, 0.6)**	0.9 (0.6, 1.2)	**0.2 (0.1, 0.4)**	**0.2 (0.1, 0.4)**	**0.1 (0.0, 0.6)**	**0.4 (0.3, 0.7)**	**0.5 (0.3, 0.7)**
2007–2008	0.7 (0.3, 1.5)	0.8 (0.4, 1.8)	0.7 (0.5, 1.0)	**0.6 (0.4, 0.9)**	0.6 (0.3, 1.1)	0.6 (0.3, 1.1)	**0.4 (0.3, 0.6)**	**0.4 (0.2, 0.6)**
2009–2012	Reference	Reference	Reference	Reference	Reference	Reference	Reference	Reference

Statistically significant associations at alpha=0.05 are shown in bold.

ART=antiretroviral treatment; BMI=body mass index; CI=confidence interval; IRR=incidence rate ratio; TB=tuberculosis.

### Factors associated with loss to follow-up

Associations of biomedical factors with loss to follow-up are less pronounced than with mortality. They included in adjusted analysis in the pre-ART cohort BAZ <−2 (adjusted IRR 3.6, 95% CI: 2.2–5.9) and in the ART cohort young age (adjusted IRR: 1.8, 95% CI: 1.1–2.9) and active TB (2.1 95% CI: 1.2–3.7). There also remained a strong protective effect of enrollment or treatment initiation before 2009 (Table 3).

### Opportunistic infections/HIV-related conditions at last visit before death

Opportunistic infections/HIV-related conditions at the last visit before a recorded death included moderate malnutrition (10 of 46 [22%] in the pre-ART cohort and 11 of 49 [22%] in the ART cohort), pulmonary TB (3 [7%] and 5 [10%]), unexplained diarrhea (3 [7%] and 5 [10%]), oral candidiasis (7 [15%] and 1 [2%]), wasting syndrome/ stunting/ severe malnutrition (3 [7%] and 2 [4%]), seborrheic dermatitis (3 [7%] and 2 [4%]), severe or recurrent bacterial pneumonia (3 [7%] and 1[2%]), and HIV encephalitis (3 [7%] and 1 [2%]). Any other opportunistic infection or AIDS qualifying symptom did not occur in more than 5% of the patients. The median time between the last visit and the data of death was 13 days (interquartile range 7–28) in the pre-ART cohort and 16 days (interquartile range 6–31) in the ART cohort.

### Reasons for missing clinic visits

Reasons for missing clinic visits (defaulting) are available for a subgroup of patients who defaulted in 2011 and had previously consented to be traced by telephone calls or home visits. Among a total of 56 children (14 [25%] pre-ART cohort and 42 [75%] on ART), 35 (63%) were contacted by telephone and 7 (13%) were visited at home. The remaining 14 (25%) children could not be reached or outcomes were not documented. Among those contacted, reasons for missing an appointment were stated as ‘traveling’ (8 [19%]), ‘absence of the parent’ (7 [17%]), ‘abandonment’ (2 [5%]), ‘forgot’ (3 [7%]), ‘work or family reasons’ (1 [2%]), and ‘other’ (21 [50%]).

### Evolution of CD4 percentage and BAZ after treatment initiation

**Fig. 2:**
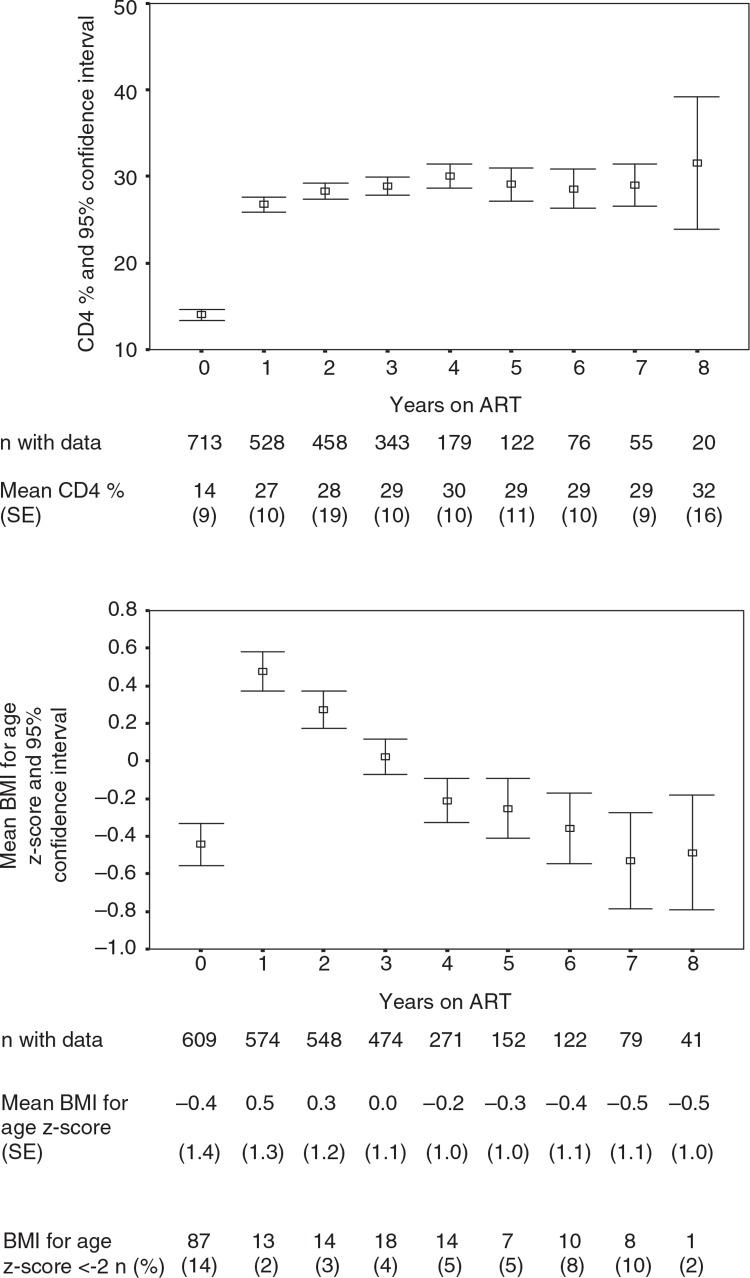
Evolution of CD4 cell counts and of BMI for age z-scores (BAZ) care of HIV-infected children on ART in the Chamanculo District of Maputo, Mozambique. Shown are mean CD4 cell counts (upper panel) and mean BMI for age z-score (BAZ) (lower panel) by year of follow-up on ART. The bars indicate 95% confidence intervals.

The mean CD4 cell percentage increased strongly from 14% (standard error [SE]: 9) at baseline to 27% (SE: 10) 1 year later. Thereafter, it increased only slightly and remained stable until 8 years of follow-up (Fig. 2).

The median BAZ increased from −0.4 (SE: 1.4) to 0.5 (SE: 1.2) within the first year after treatment initiation. It thereafter decreased again, reaching pre-ART levels at 5–6 years of follow-up. This development is paralleled by the proportion of patients who are underweight (BAZ<−2), which decreased from 14 to 2% at 1 year after treatment start and returned to 10% at 6 and 7 years of follow-up (Fig. 2).

## Discussion

To our knowledge, this is the first analysis of long-term follow-up of a pediatric HIV cohort in Mozambique and one of the longest followed cohorts worldwide (1, 8–11). The data demonstrate that good clinical and immunological outcomes are achievable at up to 8 years on ART, while retention in the pre-ART cohort was relatively poor and early increase in BAZ was subsequently lost.

The RIC at 1 or 2 years after treatment start in this study is higher than that in several previous studies from Mozambique (4–6). Reasons for this may include the slightly younger cohort in these studies or differences in the treatment models. Our results are also comparable with short-term outcomes of other international studies (11, 12), suggesting that good long-term treatment outcomes in resource-limited setting may generally be achievable and may not be specific to our setting.

In consistence with previous analysis (8), we observed that young age is one of the key factors associated with attrition. Given in addition, the relatively poor RIC of the pre-ART cohort and the relatively poor health status of the children at enrollment, as well as a high mortality in the first 3 months on treatment, our data support the recent changes in the WHO guidelines to earlier initiation of treatment of children (13).

As in other studies (8), we also found underweight to be associated with attrition. BAZ <−2 was the main risk factor for death before and after treatment initiation. In addition, malnutrition was frequently documented at the last consultation of children who subsequently died. In the absence of autopsy or hospitalization data we cannot say, whether or not malnutrition was a primary cause of death or just a consequence of underlying comorbidities. Our data, however, emphasize the importance of nutritional support for HIV-infected children (14). We also add that we used BAZ since it seemed to be an appropriate indicator for children of a large age distribution. However, there may be slightly different effects if weight for height z-scores or weight for age z-scores are used among young children, which are more suitable indicators in this age group.

Additional risk factors for death and/or loss to follow-up included active TB, underscoring the importance of TB screening, treatment and chemoprophylaxis in this context, as well as the year of enrollment/treatment initiation. The association with the year of enrollment/treatment initiation can in part be explained by the careful selection of patients by committees in the beginning of the program when treatment was limited and only few patients in need could be offered ART. It is also possible that expanding treatment options in later years increased the possibility of loss to follow-up due to unrecorded self-transfers.

After an initial sharp increase in the BAZ at 1 year on treatment, we observed a continuous decline to pre-treatment levels. A similar effect has previously been observed in a pediatric cohort from South Africa (15) and may be explained by a faster catch-up of weight than height among infants on ART. The BAZ stabilized at mean values of about −0.5 z-scores, which may be attributable to the chronic malnutrition in this and similar African populations. These declines in BAZ over time show that even among patients stably on ART, nutritional monitoring is required.

That good mid- to long-term follow-up of HIV-infected children is achievable is not self-evident since during their development children pass through various phases that can negatively impact treatment adherence. A key step in this process is the disclosure of the HIV status to the child (16). Another is the continuous support of aging caregivers, who themselves may be infected with HIV. Specific reasons for the success of our project may, therefore, include the presence of psychosocial support, in form of patient education, counseling, and defaulter tracing by lay counselors. Other medical factors that helped to achieve these results are the availability of viral load testing and the possibility to transfer children more rapidly for second-line treatment in an MSF-supported referral center.

It is noteworthy to say that the treatment outcomes were achieved even though the majority of clinical tasks were performed by lower cadre nurses and the counseling by lay counselors. These data therefore add to a growing body of literature, that shows that task shifting to lower cadre nurse and lay counselors is feasible in resource-limited settings (17) including for HIV-infected children (18, 19).

One of the strengths of this study is the use of routine monitoring data, which reflect the reality in the field. Another is that the STROBE guidelines were used for reporting of observational studies (7). Limitations include the higher number of missing data (especially for CD4 cell counts/percentages, height, weight, and WHO stage), the possibility of data errors, as well as a high probability of underreporting deaths (20), as is typical for routine data. Therefore, even measured confounders were only partially controlled in the adjusted analysis and it cannot be excluded that these results may have been influenced by information bias. The main outcomes (RIC) should, however, be unbiased and not influenced by missing values. A further limitation is that we did not fit more complex statistical models which adjust for competing risks. We also did not present regression analysis stratified by the time of event, since the number of outcomes, especially deaths was low. This should be done in future studies with larger populations.

## Conclusions

These findings justify current investments in pediatric HIV care and show that long-term RIC on ART is achievable in resource-limited settings. They also indicate that nutritional support should be an integral part of pediatric HIV programs.

## References

[1] Sutcliffe CG, van Dijk JH, Bolton C, Persaud D, Moss WJ (2008). Effectiveness of antiretroviral therapy among HIV-infected children in sub-Saharan Africa. Lancet Infect Dis.

[2] Ministério da Saúde Instituto Nacional de Saúde Maputo, Moçambique, Instituto Nacional de Estatística Maputo, Moçambique, ICF Macro Calverton, MD, EUA (2010). Inquérito Nacional de Prevalência, Riscos Comportamentais e Informação sobre o HIV e SIDA em Moçambique (INSIDA) 2009.

[3] National Directorate of Human Resources, Ministry of Health, Mozambique (2008). National Plan for Health Human Resources Development (NPHHRD) 2008–2015.

[4] Fayorsey RN, Saito S, Carter RJ, Gusmao E, Frederix K, Koech-Keter E (2013). Decentralization of pediatric HIV care and treatment in five sub-Saharan African countries. J Acquir Immune Defic Syndr.

[5] McNairy ML, Lamb MR, Carter RJ, Fayorsey R, Tene G, Mutabazi V (2013). Retention of HIV-infected children on antiretroviral treatment in HIV care and treatment programs in Kenya, Mozambique, Rwanda, and Tanzania. J Acquir Immune Defic Syndr.

[6] Lahuerta M, Lima J, Elul B, Okamura M, Alvim MF, Nuwagaba-Biribonwoha H (2011). Patients enrolled in HIV care in Mozambique: baseline characteristics and follow-up outcomes. J Acquir Immune Defic Syndr.

[7] Von Elm E, Altman DG, Egger M, Pocock SJ, Gøtzsche PC, Vandenbroucke JP (2007). The Strengthening the Reporting of Observational Studies in Epidemiology (STROBE) statement: guidelines for reporting observational studies. PLoS Med.

[8] Bolton-Moore C, Mubiana-Mbewe M, Cantrell RA, Chintu N, Stringer EM, Chi BH (2007). Clinical outcomes and CD4 cell response in children receiving antiretroviral therapy at primary health care facilities in Zambia. JAMA.

[9] Leroy V, Malateste K, Rabie H, Lumbiganon P, Ayaya S, Dicko F (2013). Outcomes of antiretroviral therapy in children in Asia and Africa: a comparative analysis of the IeDEA pediatric multiregional collaboration. J Acquir Immune Defic Syndr.

[10] Kabue MM, Buck WC, Wanless SR, Cox CM, McCollum ED, Caviness AC (2012). Mortality and clinical outcomes in HIV-infected children on antiretroviral therapy in Malawi, Lesotho, and Swaziland. Pediatrics.

[11] Sauvageot D, Schaefer M, Olson D, Pujades-Rodriguez M, O'Brien DP (2010). Antiretroviral therapy outcomes in resource-limited settings for HIV-infected children <5 years of age. Pediatrics.

[12] Marazzi MC, De Luca S, Palombi L, Scarcella P, Ciccacci F, Ceffa S (2014). Predictors of adverse outcomes in HIV-1 infected children receiving combination antiretroviral treatment: results from a DREAM Cohort in Sub-Saharan Africa. Pediatr Infect Dis J.

[13] World Health Organization, UNAIDS (2013). Consolidated guidelines on the use of antiretroviral drugs for treating and preventing HIV infection: recommendations for a public health approach.

[14] Sunguya BF, Poudel KC, Otsuka K, Yasuoka J, Mlunde LB, Urassa DP (2011). Undernutrition among HIV-positive children in Dar es Salaam, Tanzania: antiretroviral therapy alone is not enough. BMC Public Health.

[15] Shiau S, Arpadi S, Strehlau R, Martens L, Patel F, Coovadia A (2013). Initiation of antiretroviral therapy before 6 months of age is associated with faster growth recovery in South African children perinatally infected with human immunodeficiency virus. J Pediatr.

[16] Hazra R, Siberry GK, Mofenson LM (2010). Growing up with HIV: children, adolescents, and young adults with perinatally acquired HIV infection. Annu Rev Med.

[17] Kiweewa FM, Wabwire D, Nakibuuka J, Mubiru M, Bagenda D, Musoke P (2013). Noninferiority of a task-shifting HIV care and treatment model using peer counselors and nurses among Ugandan women initiated on ART: evidence from a randomized trial. J Acquir Immune Defic Syndr.

[18] Cohen R, Lynch S, Bygrave H, Eggers E, Vlahakis N, Hilderbrand K (2009). Antiretroviral treatment outcomes from a nurse-driven, community-supported HIV/AIDS treatment programme in rural Lesotho: observational cohort assessment at two years. J Int AIDS Soc.

[19] Monyatsi G, Mullan PC, Phelps BR, Tolle MA, Machine EM, Gennari FF (2012). HIV management by nurse prescribers compared with doctors at a paediatric centre in Gaborone, Botswana. S Afr Med J.

[20] Van Cutsem G, Ford N, Hildebrand K, Goemaere E, Mathee S, Abrahams M (2011). Correcting for mortality among patients lost to follow-up on antiretroviral therapy in South Africa: a cohort analysis. PLoS One.

